# Magnetic Particle Filled Elastomeric Hybrid Composites and Their Magnetorheological Response

**DOI:** 10.3390/ma11061040

**Published:** 2018-06-19

**Authors:** Seung Hyuk Kwon, Jin Hyun Lee, Hyoung Jin Choi

**Affiliations:** 1 Department of Polymer Science and Engineering, Inha University, Incheon 22212, Korea; 22141016@inha.edu; 2 Polymer Technology Institute, Sungkyunkwan University, Suwon 16419, Korea

**Keywords:** magnetorheological elastomer, magnetic particle, viscoelastic, damping property

## Abstract

The magnetorheological (MR) elastomer as a hard and soft hybrid functional material, a composite material consisting of magnetic hard particles embedded in elastomeric soft matrix, is a branch of MR materials that are functional smart materials rapidly responding to external magnetic fields. These tunable properties of MR elastomers facilitate a variety of applications. In this brief review paper, in addition to general information on the MR elastomers, recent research not only on a wide variety of MR elastomeric systems focusing on various magnetic particles, elastomeric matrices, additives and particle modification methods, but also on their characteristics including MR properties from dynamic oscillation tests is covered along with their mechanical properties such as the Payne effect, tensile strength and engineering applications.

## 1. Introduction

Recently, world-wide interest in smart and intelligent materials has shown huge growth [1,2,3]. Smart materials are materials that can change their material properties in response to applied external stimuli, such as electric or magnetic fields, stress, pH, moisture, temperature, light, and so on [4].

Magnetorheological (MR) materials belonging to functional smart materials exhibit tunable rheological and viscoelastic characteristics such as shear stress, yield stress, dynamic modulus and damping when regulated by an applied external magnetic field, so-called MR phenomenon first discovered in the late 1940s [5,6,7,8,9]. In general, a wide range of MR materials has been categorized as MR fluid, MR elastomer, MR gel, MR grease and MR foam, mainly depending on the phase matrix materials used [10]. MR materials consist of magnetic particles incorporated in a non-magnetic medium. The most applicable one among the MR materials is considered to be the MR fluid since it produces the highest MR effect based on the rheological properties measured [11]. The yield stress and shear viscosity of the MR fluids increase by many orders of magnitude, and the suspension system changes from a Newtonian liquid to a solid-like state when a magnetic field is applied [12,13]. Their variety of applications includes magnetically-controllable devices such as brakes, clutches, dampers and mounts for semi-active or adaptive vibration controlling [14,15,16,17,18]. However, despite their good performance and several successful commercial applications, MR fluids exhibit distinct shortcomings such as deposition, sedimentation, environmental contamination and sealing problems [19,20,21]. These disadvantages tend to limit the huge growth of their wider engineering applications [22]. Concurrently, MR gels, another system with an intermediate state between the states of MR fluid and MR elastomer, have been also introduced. Various studies including tunable viscoelasticity of magnetic MR gels have been reported [23,24,25,26,27].

On the other hand, MR elastomer, one of the hard and soft hybrid functional and smart materials, is considered to be an alternative material to overcome the shortcomings of the MR fluids. The MR elastomers are solid and can be used for stiffness-controllable devices unlike MR fluids being used in viscosity-controllable devices. In addition, they are composed of nano- or micron-sized hard magnetic particles dispersed in an elastomeric soft matrix. The magnetic particles used in MR elastomers are carbonyl iron, iron oxides and other soft-magnetic particles without magnetic hysteresis, and suitable elastomer matrix materials include natural rubber [28,29], silicone rubber [30,31,32], nitrile rubber [33], polybutadiene rubber [34,35] and polyurethane rubber [36,37]. Specifically, magnetically-controllable elastomer composites with magnetic particles promise to have more functions than conventional elastomers. MR elastomers can be applied for automotive bushings, engine mounts and adaptively-tuned vibration absorbers [38,39,40,41,42,43]. The first comprehensive investigation of MR elastomers was conducted by Jolly et al. [44], in which a quasi-static dipolar mechanism was introduced to interpret the dynamic modulus change of an MR elastomer. Based on all these merits, MR elastomers have drawn huge attention and have become an emerging research topic. In addition, related to their engineering applications, many theoretical models have been also extensively introduced in attempts to characterize the mechanical behaviors of the MR elastomers [45,46,47]. Recently, Cantera et al. [48] reviewed models for predicting the magneto-mechanical response of MR elastomers under various loads.

MR elastomers can be at first classified into two kinds: isotropic MR elastomers and anisotropic MR elastomers. The former is characterized by having a uniform magnetic particle distribution in the matrix, and the latter has a special chain-like structure of magnetic particles in a matrix resulting from curing the matrix under an applied magnetic field. It has been known that the MR effect of anisotropic MR elastomers is larger than that of isotropic ones at the same particle content [49]. Jung et al. [50] investigated the MR performance of isotropic and anisotropic MR elastomer systems prepared using natural rubber (NR) and carbonyl iron (CI) particles and found that the anisotropic MR elastomer possessed a larger storage modulus than the isotropic one, which was explained as due to the reason that the chain-like structure formed by aligned particles along the field direction acts as a rod-like filler. Similarly, Lu et al. [51] reported that for MR elastomers consisting of thermoplastic poly(styrene-b-ethylene-co-butylene-b-styrene) rubber and CI particles, the anisotropic MR elastomer showed an even higher initial storage modulus because the filler effect resulting from the chain-like structure of the particles enhanced the magnetic permeability of the MR elastomer.

This article briefly reviews recent research on the fabrication and characterization of various hard and soft hybrid functional MR elastomers along with their physical and mechanical properties and potential industrial applications. Specifically, their viscoelastic characteristics can be modified by not only external stimuli such as an external magnetic field, stress and temperature, but also their intrinsic features determined by the constitution of components, network structure, crosslinking density, the type and size of particles, and so on. In particular, the microstructures with either the isotropic or anisotropic state of the MR elastomers were confirmed by using scanning electron microscopy (SEM), while the magnetic properties of hard magnetic particles were measured by vibrating-sample magnetometer (VSM) analysis. The viscoelastic MR properties of MR elastomers such as dynamic moduli and loss tangent and MR efficiency were observed by a rotation-typed rheometer attached to a magnetic field generator. Furthermore, mechanical properties of MR elastomers such as the Payne effect and damping factor are also discussed along with their extensive potential engineering applications.

## 2. Magnetorheological (MR) Elastomer

Structurally, the MR elastomers can be thought of as solid analogs of MR fluids, which are mainly composed of soft-magnetic magnetic particles suspended in non-magnetic fluids. However, there are some differences in the way in which these two limiting classes of materials are typically intended to operate. Noteworthy is that the magnetic particle chains within the elastomer matrix are intended to function in the pre-yield region, while MR fluids typically activate within a post-yield continuous shear or flow regime. Therefore, the efficiency of the MR fluids is explained by their field-dependent yield stress, while the strength of MR elastomers is generally analyzed by their field-dependent dynamic modulus [10].

**Figure 1:**
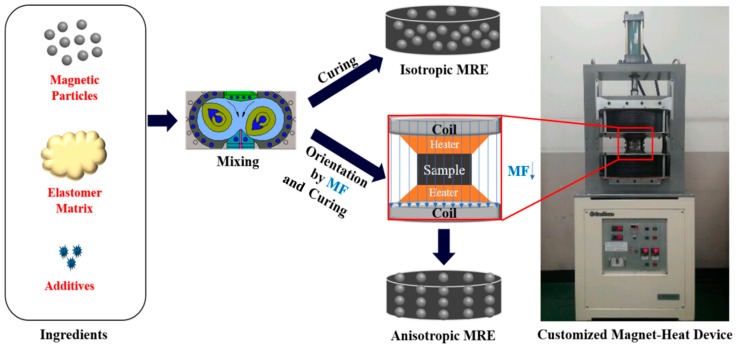
Schematic diagram of fabrication process of isotropic and anisotropic magnetorheological elastomers (MRE).

The fabrication process of MR elastomers is similar to that of conventional rubber. The material ingredients are magnetic particles, elastomer matrix and additives. The fabrication process of MR elastomers consists of three steps: mixing, curing and magnetic particle orientation, as shown in Figure 1. After all ingredients are mixed in an internal mixer under a sufficient processing temperature, the isotropic MR elastomers are cured without the presence of a magnetic field, while the anisotropic MR elastomers are cured with the action of a magnetic field. For instance, the anisotropic MR elastomer of rubber/CI particles was prepared in a magneto-heating coupled device in the presence of a magnetic field (indicated as arrows in Figure 1) [50]. The curing process for anisotropic MR elastomers requires a strong magnetic field (MF), usually above 0.8 T, to form the chain-like structure of dispersed magnetic particles in the rubber matrix following the magnetic field direction. A constant temperature is required to maintain the flexibility of the magnetic particles for both isotropic and anisotropic MR elastomers during curing [52].

### 2.1. Magnetic Particles

Generally, soft-magnetic particles are considered as favorable candidates for MR materials because of their appropriate magnetic properties including simple magnetization and demagnetization, almost no magnetic hysteresis and a large magnetization saturation constant [53]. The high magnetic permeability of the particles easily attracts small magnetic leakage fields in the material compound, thus inducing the maximum possible MR effect [54]. CI [55,56,57], iron oxide [58,59,60,61] and nickel [62] have been utilized as the magnetizable particles in MR materials. Among these magnetic particles, the CI particles have been most commonly used not only because of their excellent magnetic properties with high permeability and low remnant magnetization, but also due to their spherical shape. Consequently, many research works regarding MR elastomers have focused on studying the influence of CI particles on the rheological behavior of the MR elastomers.

Several groups have also reported the effects of magnetized nanoparticles of CoFe_2_O_4_ [63], Ni [64] and FeCo_3_ [65] included in MR elastomers (i.e., silicone rubber) and Fe [66] incorporated in MR suspensions (or MR elastomers) on the MR elastomer composites.

Chen et al. [67] prepared MR elastomers with nitrile butadiene rubber (NBR) and Fe_3_O_4_ with high mechanical properties using zinc dimethacrylate (ZDMA). The polymerized ZDMA during the NBR vulcanization caused the zinc ion paring to NBR and the interaction with Fe_3_O_4_, resulting in excellent mechanical properties and magnetic properties. However, it should not be ignored that addition of ZDMA also showed bad chemical stability to acid and base. Kurniawan et al. [68] also investigated Fe_3_O_4_ particles coated with polyethylene glycol, which could be a promising candidate for the silicone rubber-based MR elastomer due to their good mechanical and magnetic characteristics and also a desirable candidate as microwave absorbing materials. Furthermore, Mietta et al. [69] synthesized Fe_3_O_4_/Ag nanoparticles by using the co-precipitation method for new magnetic MR elastomers. Besides, some researchers have studied nickel particles for the MR elastomer systems. Landa et al. [70] synthesized nickel nanoparticles and nanochains, then used them as fillers for the fabrication of the anisotropic MR elastomer with the silicone rubber matrix.

The magnetic particles coated with an organic or inorganic material via a modified Stöber method were observed to enhance not only the sedimentation properties of MR fluids, but also the anti-acidic and anti-oxidation resistance of the dispersed magnetic particles [71,72]. Therefore, in the case of MR elastomers, such a coating technique could be utilized to prevent the particles from oxidation and improve their compatibility with the elastomeric matrix [73]. Actually, the hydrophilic properties of the CI particles make them incompatible with the hydrophobic elastomer matrix. Consequently, such magnetic particles may de-bond from the elastomer matrix, leading to the deterioration of the mechanical properties of the MR elastomer.

Recently, some studies to overcome this problem by fabricating the magnetic particles being surface-modified with organic or inorganic materials have been reported by using proper compatibilizers [74,75,76,77,78,79]. Li et al. [75] prepared core-shell structured poly(methyl methacrylate) (PMMA)-coated CI particles to investigate the effect of particle coating on the dynamic properties of the MR elastomers with PMMA matrix. Compared to the non-coated CI particles, the use of PMMA-coated CI particles yielded a lower MR effect; however, they produced a weak Payne effect and a small steady loss factor. Fuchs et al. [76] fabricated surface-coated iron particles using poly(fluorostyrene) for the silicone rubber-based MR elastomers. It was found that the MR elastomers with surface-coated iron particles possessed superior mechanical properties with respect to the oxidation stability test. Behrooz et al. [77] prepared poly(tetrafluoropropyl methacrylate) (PTFPMA)-coated iron particles utilizing a combination of reversible addition fragmentation chain transfer polymerization and click chemistry techniques. The PTFPMA-coated iron particles did not show a significant change in the shear modulus of the MR elastomers, but the loss of shear modulus due to oxidation was reduced. Maleic anhydride was also selected as the compatibilizer to modify the interface of MR elastomers to improve their damping properties [78]. The compatibility between the magnetic particles and rubber matrix could be enhanced by increasing the content of the compatibilizer. The enhancement of the bond between the two components (particles and matrix) in the MR elastomers produced the superior mechanical properties of the loss factor and tensile strength. Furthermore, An et al. [79] fabricated (3-aminopropyl) triethoxy silane (APTES)-coated CI particles in order to have better affinity with natural rubber matrix. The silane-coated CI particle-embedded MR elastomers have improved mechanical properties and MR effect.

Recently, the majority of published works have been focused on the investigations of the rheological properties of MR elastomers with monodispersed magnetic particles, although MR elastomers with a bimodal distribution of particles have also received some attention. Especially, the combinations of magnetic and nonmagnetic particles or two types of magnetic particles have been considered as fillers of MR elastomers. Li et al. [80] and Sorokin et al. [81] investigated the MR properties of two different MR elastomers filled with various proportions of micron-sized iron and nano-sized magnetite particles. The MR elastomers with mono-modal particles showed a higher MR effect.

### 2.2. Elastomer Matrix

Various elastomers can be used as matrices to fabricate MR elastomers with different properties. The elastomer matrix plays an important role in the mechanical performance of MR elastomers, and the selection of a suitable matrix material is regarded to be significantly important when we need to consider the possible applications and long-term stability of the MR elastomeric composites. Furthermore, based on the stiffness of elastomeric matrices, their softness could be changed from soft to hard. Among the MR elastomeric matrices mentioned above [82], silicone rubber and natural rubber are two kinds of the most common matrices reported in MR elastomer research [83]. For the use of silicone rubber, there are many advantages such as its simple processing caused by liquid precursors with low shear viscosity and its higher relative MR effect. Furthermore, it was reported that silicone rubber is more resistant to heat, chemicals, fungi, UV and O_3_ compared to natural rubber (NR) [84]. However, this kind of soft matrix could not satisfy the needs of applications of high impact loading [85]. Perales-Martinez et al. [86] studied how different contents of CI particles embedded in a silicone rubber affect the MR response of the MR elastomers. Li et al. [87] investigated the viscoelastic properties of both isotropic and anisotropic MR elastomers with silicone rubber as the matrix by using compression and shear methods in frequency and strain swept tests.

On the other hand, NR is also considered to be more suitable for an MR elastomer matrix material especially for practical engineering applications than other rubbers due to its superior mechanical properties [88]. Chen et al. [89] fabricated NR-based MR elastomers and found that their mechanical properties in terms of tensile/tear strength and hardness are better than those of silicone rubber-based MR elastomers. As examined by Li et al. [90], the NR-based MR elastomers have also shown the capability to generate a relatively higher MR effect and magneto-induced storage modulus.

Nonetheless, it is known that NR exhibits poor aging, weathering and resilience over a wide temperature range, as well as relatively low resistance to oil [91]. These drawbacks somewhat limit the applications of the NR-based MR elastomers. As a partial solution for these drawbacks, it is well known that epoxidized natural rubber (ENR), prepared in carefully-controlled conditions and classified as a biodegradable and eco-friendly material since it is produced from a renewable natural resource, demonstrates some new and unique properties beyond the common features of NR in terms of damping ability, gas permeability, oil resistance and rolling resistance [92]. These properties are very critical for MR elastomer applications, particularly for various devices in automobile and mechanical industries.

It was also reported that the mechanical properties of silicone rubber and the degradation stability of natural rubber were poor compared to polyurethane (PU) elastomers [93]. In the study, PU rubber was selected as a matrix because of its better degradation stability than NR and superior mechanical stability than silicone rubber. The properties of PU rubbers such as tensile strength, stiffness, friction coefficient and chemical resistance can be easily adjusted by altering the types of soft and hard segments and the content of the hard segments [94]. Wei et al. [95] prepared PU rubber-based MR elastomers, and their mechanical properties, including shear modulus, MR effect, loss factor and glass transition temperature, were characterized with a dynamic mechanical analyzer. In addition, Wu et al. [96] prepared anisotropic MR elastomers composed of polytetramethylene ether glycol-based polyurethane rubber and iron particles. The difficulty in the orientation of iron particles in the polyurethane matrix was overcome by ball mill mixing.

### 2.3. Additives

Many reported studies of MR elastomer systems involved in some additives, which are used as parts of the MR elastomer ingredients. The types of additives include a reinforcing agent, magnetic filler, plasticizer, conductive material and crosslinking agent. These are typically added to increase the performances of MR elastomers. The additives most used in MR elastomers are classified into two categories. The first one is a mechanical reinforcing filler such as carbon black, silicon carbide, graphite, graphene and carbon nanotubes (CNTs) and is utilized to improve the mechanical properties of MR elastomers. Chen et al. [97] and Nayak et al. [98] investigated the isotropic MR elastomers fabricated with and without the addition of carbon black into the matrix material and found that the addition of carbon black improved the mechanical properties of the MR elastomers. Tian et al. [99] fabricated both isotropic and anisotropic MR elastomers with various weight fractions of graphite and found that the graphite particles increased the initial mechanical properties and decreased the MR effect. Silicon carbide (SiC) particles were also introduced in order to enhance the dynamic mechanical performance of the MR elastomers [100], while the SiC particles have been broadly used in rubber technology because of their great bonding with the rubber matrix, and they can highly improve the performance and mechanical properties of rubber material. Bica et al. [101] utilized graphene nanoparticles to produce a hybrid electro-conductive MR elastomer for a magnetoresistive sensor. Recently, it has been reported that CNTs act as a filler for the fabrication of MR elastomers, providing enhanced dynamic mechanical and MR properties [102,103]. Aziz et al. [103] added functionalized multiwall CNTs into the MR elastomers prepared with NR and carbonyl iron particles and found that the addition of the CNTs contributed to the formation of the interconnected network of NR, resulting in the enhanced MR and mechanical properties of MR elastomers.

The magnetic fillers have a considerable influence on the MR properties of the MR elastomer composites. Davis et al. [104] have theoretically found that the optimum volume percent of magnetic fillers for increasing the shear modulus was 27 volume percent. However, the MR effect of the MR elastomer composites is quite low for practical industrial applications. Stepanov et al. [105] and Kramarenko et al. [106] synthesized MR elastomers composed of silicone rubber with carbonyl iron particles and hard magnetic filler particles (NdFeB) and investigated their viscoelastic properties depending on the size and concentration of filler particles in the MR elastomers. The deformation of the MREs was controlled by the applied magnetic field, and their elastic and loss moduli, depending on strain, increased up to more than 100-times. Finally, adding certain kinds of plasticizers significantly improved the MR effect of MR elastomers. Lokander et al. [107] investigated the relative MR effect of the isotropic MR elastomers with nitrile rubber and iron particles at the critical particle volume concentration at a high magnetic field (~0.8 T) and found that their relative MR effect was increased by adding di-2-ethylhexylphthalate as a plasticizer. Wu et al. [108] showed that that diisooctyl phthalate (DOP) used as a plasticizer also enhanced both the absolute and relative MR effects of PU rubber-based MR elastomers, and the DOP particles incorporated into the PU rubber matrix not only softened the matrix, but also improved the MR effect.

## 3. Characterizations of MR Elastomer

### 3.1. Morphology

**Figure 2:**
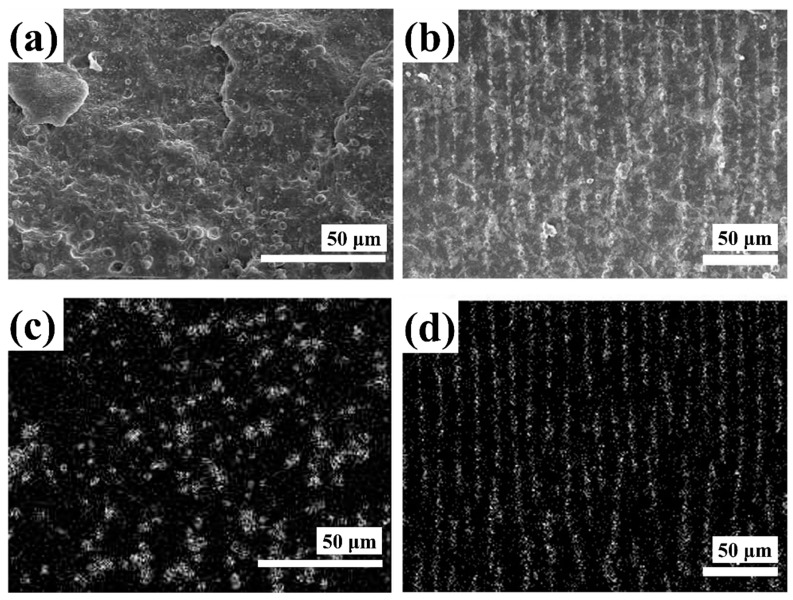
SEM and Fe-mapping images of the pure carbonyl iron (CI) particles incorporated in the natural rubber (NR)-based isotropic (**a**,**c**) (reprinted with permission from [50], Copyright 2016 Elsevier B. V., New York, NY, USA) and anisotropic (**b**,**d**) MR elastomers (reprinted with permission from [79], Copyright 2017 Elsevier B. V., New York, NY, USA).

The microstructures of MR elastomers mainly regarding the dispersion of magnetic particles in the matrix have been observed by SEM [50,79]. Typically, prior to the SEM observations, first MR elastomer samples were cut perpendicularly to the surface of the disc, after immersing the samples in liquid nitrogen. Through the observations of the structural images, interfacial adhesion between the rubber matrix and dispersed magnetic particles could be seen. In addition, the alignment of magnetic particles within the rubber matrix was characterized using the energy dispersive X-ray spectrum (EDAX) with Fe-mapping image analysis. Figure 2 shows the microstructure of pure CI particles dispersed in NR with/without magnetic fields. Figure 2a displays the randomly-dispersed CI particles in an NR matrix from the SEM images of the isotropic MRE prepared in the absence of a magnetic field. Its Fe-mapping image of EDAX analysis, in which the bright dots are considered to be the CI particles dispersed in the continuous NR matrix, is presented in Figure 2c. On the other hand, Figure 2b,d shows, respectively, the SEM images and EDAX mapping image of the morphologies of the anisotropic MR elastomer samples. Figure 2d shows that the Fe contents from the CI particles exist, and the CI particles in the matrix are aligned clearly with the applied magnetic field direction during the curing process. These morphological states of the MR elastomers are strongly related to their MR characteristics together with their potential engineering applications. Recently, for a better understanding of the microstructural formation of the embedded magnetic particles in the MR elastomers, X-ray micro-computed tomography (CT) [109,110] was adopted, and the characterization by utilizing CT provided a three-dimensional map of the structure and geometry of the samples, while preserving the pristine structure.

### 3.2. Magnetic Property

Magnetic properties of both soft-magnetic particles adopted and MR elastomers have been characterized by the investigation of their magnetization and hysteresis loop measurements [76,100].

**Figure 3:**
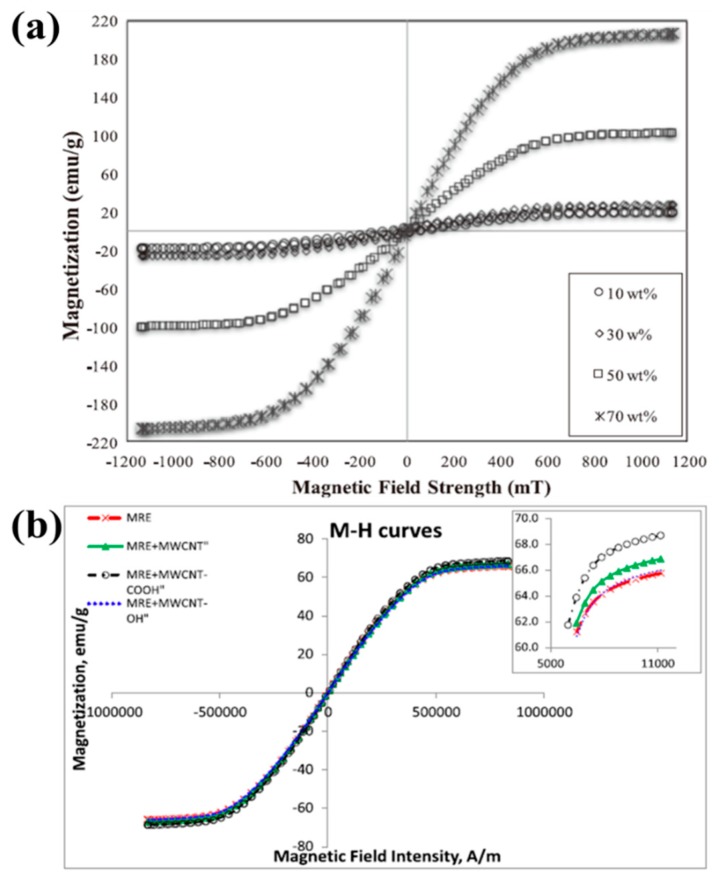
Magnetization curves of (**a**) the epoxidized NR (ENR)-based MR elastomers for different contents of CI particles (reprinted with permission from [85], Copyright 2016 IOP Publishing Ltd., Bristol, UK) and (**b**) the MR elastomers samples containing CNTs (reprinted with permission from [103], Copyright 2016 IOP Publishing Ltd., Bristol, UK).

Figure 3a illustrates the magnetization curves of the epoxidized NR (ENR)-based MR elastomers for different contents of CI particles [76]. In the case of all samples, the magnetization curves reveal narrow magnetic hysteresis loops. This fact indicates that the ENR-based MR elastomers exhibit a soft magnetic characteristic. The trend of the graphs is similar for all samples in such a way that the magnetization curves were increased dramatically up to a certain magnetic field intensity, approximately 400 mT and 600 mT.

Figure 3b shows the hysteresis loops of the various MR elastomers with or without CNTs (pure MR elastomer, MR elastomer with CNTs, MR elastomer with carboxylated CNTs (COOH-MWCNT) and hydroxylated CNTs (OH-CNT), measured in magnetic fields up to 1.2 T [94]. Overall, the MR elastomer samples containing CNTs have a higher magnetic saturation value (*M_S_*) compared to those without CNTs. According to Fang et al. [111], a possibly reason is that the CNTs used as an additive strengthen the chain-like and columnar structure formed by CI particles and CNTs, resulting in the higher saturation magnetization.

## 4. MR Characteristics

### 4.1. Dynamic Test

MR elastomers are known as viscoelastic materials that can store and dissipate some of the energy during deformation under both external shear and magnetic fields. Generally, the ability of the material to store energy elastically is related to the storage modulus (G′), which represents the elasticity of the material. On the other hand, the loss modulus (G″), which quantifies the ability to dissipate the energy of deformation as heat, represents the viscous properties of the material. Fundamentally, G′ and G″ are principal parameters describing the rheological properties of viscoelastic materials, as well as MR materials, including MR elastomers. To examine the MR characteristics of MR elastomers, both the strain amplitude sweep test and angular frequency sweep test are typically used using a rotational rheometer equipped with the magnetic field generator and the disk-type MR elastomer samples.

**Figure 4:**
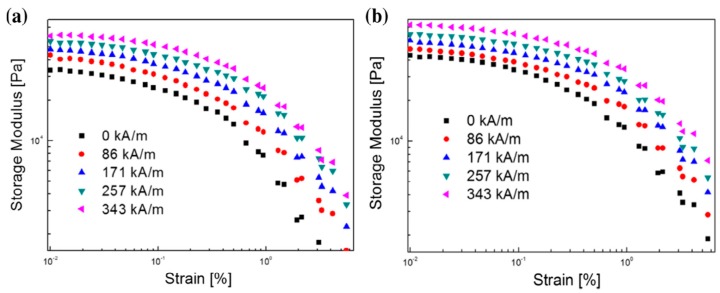
Strain amplitude sweep curves of the NR-based MR elastomers under various magnetic field strengths: (**a**) isotropic MR elastomers and (**b**) anisotropic MR elastomers (reprinted with permission from [50], Copyright 2016 Elsevier B. V., New York, NY, USA).

The strain amplitude sweep test is used to define the linear viscoelastic (LVE) region for a frequency sweep test as the next step. Once the LVE region limit is determined, the dynamic properties of the MR elastomer could be studied within the linear region and in the nonlinear region, separately, to show the influence of the LVE limit on the dynamic properties. Figure 4a,b shows the storage moduli of the isotropic and anisotropic MR elastomers composing of NR and CI particles, respectively, as a function of strain for different magnetic field strengths (in the applied strain range 0.01–5% and at a fixed frequency of 1 Hz), measured in order to obtain the position of the LVE region. From both figures, it is determined that the storage modulus increases with increasing magnetic field strength. In addition, overall, the storage modulus decreases with increasing strain amplitude, reducing rather slowly within a 0.1% strain and then sharply over a 0.1% strain. The storage moduli of the two different MR elastomer samples maintain constant plateau values at the low strain region and then decrease gradually with increased strain. As expected, in the LVE region, the storage moduli are independent of the applied strain. In addition, the storage moduli of the anisotropic MR elastomers are shown to be higher than those of the isotropic MR elastomers at the same magnetic field strength.

**Figure 5:**
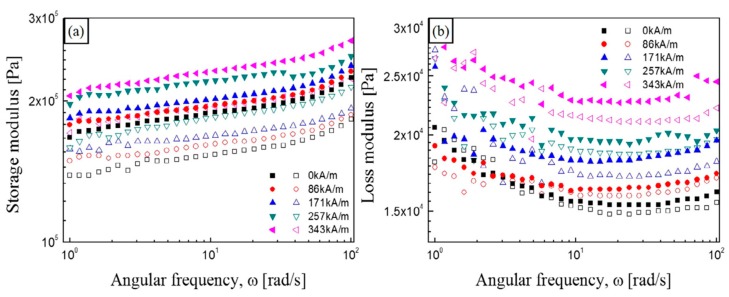
Frequency sweep test: storage modulus (**a**) and loss modulus (**b**) as a function of angular frequency for the anisotropic NR-based MR elastomers under various magnetic field strengths. (closed symbols: CI/(3-aminopropyl) triethoxy silane (APTES) MR elastomers; open symbols: pure CI MR elastomers) (reprinted with permission from [79], Copyright 2017 Elsevier B. V., New York, NY, USA).

Figure 5a,b presents the frequency dependency of the storage moduli and loss moduli, respectively, of (3-aminopropyl) triethoxy silane (APTES)-coated CI-based MR elastomers and pure CI-based MR elastomers in a range of angular frequencies from 1–100 rad/s at a mean strain value of LVE [79]. With increasing magnetic field strength, all the anisotropic NR-based MR elastomer samples including CI or CI/APTES particles exhibit an enhancement of their storage moduli. In addition, the storage moduli of the ATPES-coated CI-based MR elastomer (closed) are seen to be higher than those of the pure CI-based MR elastomer (open).

**Figure 6:**
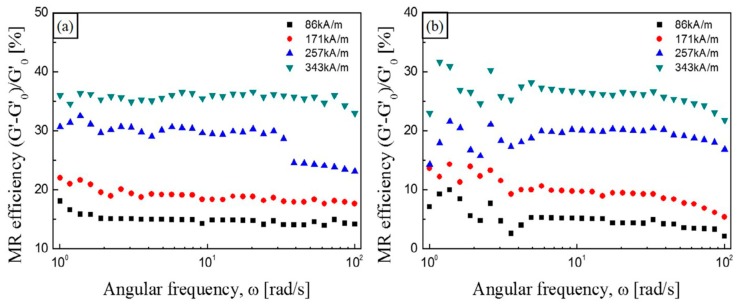
Relative MR effect of (**a**) CI/APTES MR elastomer and (**b**) CI MRE as a function of angular frequency under various magnetic field strengths (reprinted with permission from [79], Copyright 2017 Elsevier B. V., New York, NY, USA).

Fundamentally, the MR effect, one of the key parameters for evaluating the performance of MR elastomers, is usually calculated by storage moduli. The MR effect can be described by both absolute and relative MR effects. The absolute MR effect (ΔG′) is the difference between the maximum storage modulus (G′_max_) achieved in the presence of a magnetic field and the shear modulus obtained without a magnetic field (G_0_). The absolute MR effect is expressed by the following equation: ΔG′ = G′_max_ − G_0_, and the relative MR effect equation is represented as follows:

(1) $$\;\mathrm{Relative}\;\mathrm{MR}\;\mathrm{Effect}=\;\frac{G^{\prime }-G_{0}}{G_{0}}\times 100,$$

where $G_{0}$ is the initial storage modulus without a magnetic field and $G^{\prime }$ is the magneto-induced storage modulus at the magnetic field strength. Through dynamic mechanical tests, the MR effect of MR elastomers is shown to be dependent on the frequency and strain amplitude. Figure 6 shows the MR effect of CI/APTES-based MR elastomers (a) and pure CI-based MR elastomers (b) as a function of frequency [79]. At all magnetic field strengths, the MR effect of the CI/APTES MR elastomers is higher than that of the pure CI MR elastomers.

### 4.2. Creep Test

The phenomenon of creep often appears when the strain of materials changes over time slowly under a constant stress. If the stress is instantaneously removed, the dependence of strain on time is defined as the recovery behavior, and it is believed that the information of the creep and recovery behavior of a MR elastomer can provide the guidance for its engineering applications. Due to its importance in understanding the viscoelastic behaviors of the systems, it has been widely applied not only for polymeric materials, but also smart materials such as electrorheological suspensions and magnetic fluids [112,113,114]. However, there are merely a few reports on the creep and recovery behavior of MR elastomers. Li et al. [115] found that the response strain of MR fluids was highly dependent on the constant stress level and proposed a thick column structure hypothesis to explain the creep behaviors of MR fluids. In addition, both experimental and modeling studies of the creep and recovery behaviors of MR elastomers were investigated. A four-parameter viscoelastic model was developed to describe the creep behaviors of MR elastomers. The results indicated that the model can predict the creep behaviors of MR elastomers very well [116]. Xu et al. [117] also systematically investigated the creep and recovery behaviors of MR elastomers prepared with PU/epoxy resin (EP) interpenetrating polymer networks. They reported that the creep and recovery behaviors of MR elastomers are influenced by the magnetic field, particle distribution and temperature. Yu et al. [118] tested the compression creep performance of PU-based MR elastomers. To understand the mechanism of the time-dependent mechanical properties and improve the properties of MR elastomers for an engineering application, deeper research into the creep and recovery behavior of MR elastomers is needed.

**Figure 7:**
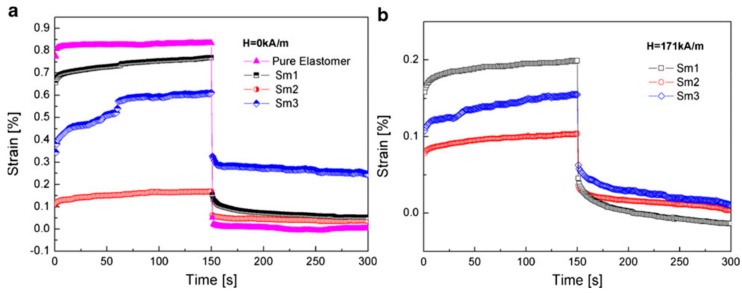
Creep and recovery curves of pure elastomer and the MR elastomer samples without (**a**) and with (**b**) the stimuli of a magnetic field (171 kA/m). Applied stress is 30 Pa (reprinted with permission from [119], Copyright 2012 Springer-Verlag, Berlin, Germany).

Bica et al. [119] investigated the creep behavior of silicone-based MR elastomers containing carbonyl iron (CI) particles, in which the MR elastomer samples used for the investigation were named according to the weight fraction of silicone rubber and MR suspension (CIs in silicone oil): Sample 1 (Sm1) (75% silicone rubber and 20% MR suspension), Sm2 (55% silicone rubber, 40% MR suspension) and Sm3 (35% silicone rubber, 60% MR suspension). As shown in Figure 7a,b, as soon as the constant stress of 30 Pa is applied, an instantaneous deformation and a nearly total instantaneous recovery of MR elastomer samples are observed without any apparent delayed response. In the absence of a magnetic field (Figure 7a), the elastomers show delayed strain following decreased instantaneous strain in both creep and recovery processes due to the negative effect of the CI particles on the elasticity of the elastomer matrix. When measured in the presence of a magnetic field (Figure 7b), all the samples exhibit a much reduced instantaneous strain under the impact of the same stress as the anisotropic structures of particles enhance the solid properties of the elastomers under the magnetic field strength. Note that the Sm2 sample showed the highest solid properties with and without an external magnetic field.

## 5. Mechanical Properties

### 5.1. Payne Effect

**Figure 8:**
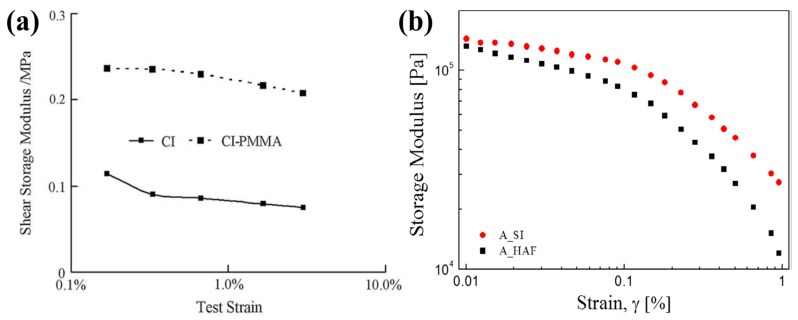
The storage moduli of (**a**) CI/PMMA- (dotted) and pure CI- (solid) based MR elastomers (reprinted with permission from [75], Copyright 2009 Elsevier B. V., New York, NY, USA) and (**b**) CI/APTES- (A_SI, circle) and pure CI- (A_HAF, square) based MR elastomers (reprinted with permission from [79], Copyright 2017 Elsevier B. V., New York, NY, USA).

The Payne effect is the phenomenon of softening stress at small strain for of a rubber filled with particles, named after the British rubber scientist, Payne [120]. It is measured under a cyclic loading condition with a small strain amplitude, considering the dependence of the storage modulus on the applied strain amplitude. Many researchers studied the Payne effect of anisotropic and isotropic MR elastomers and found that MR elastomers have a much larger storage modulus at low strains than at high strains [121]. The Payne effect is defined as the ratio of the change from initial to infinite modulus values to the initial modulus value of the material. The Payne effect can be calculated from the following Equation (2);

(2) $$\;\mathrm{Payne}\;\mathrm{Effect}=\;\frac{\left(G_{0}^{\prime }-G_{\infty }^{\prime }\right)}{G_{0}^{\prime }},$$

where $G_{0}^{\prime }$ and $G_{\infty }^{\prime }$ are the values of the storage modulus at initial and infinite strain, respectively. Figure 8 shows the G′ values as a function of strain for surface-coated CI-based MR elastomers and non-coated CI-based MR elastomers, representing the Payne effect based on the fact that the storage modulus decreases steadily with the increase of strain in the composite rubber [75,79]. The storage moduli of the surface-coated CI-based MR elastomers are much larger than those of the pure CI-based MR elastomers over the full strain range. The surface-coated CI-based MR elastomers have a smaller Payne effect than the pure CI-based MR elastomer due to increased bond strength between the particles.

The Payne effect, which has been widely adopted for analyzing rubber-polymer networks, has been recently also applied to both MR elastomers and polymer blends. The term “magnetic field-enhanced Payne effect” was first introduced by An et al. [122] for the MR gels to highlight their strain-softening. Recently, Sorokin et al. [123] also showed that the Payne effect in silicone rubber-based MR elastomers including magnetic fillers was enhanced by applying the magnetic field. Both the absolute values of the dynamic moduli and magneto-induced Payne effect were increased with the increment of the content of magnetic filler incorporated within the elastomer, which consequently increased the contribution from magnetic interactions.

### 5.2. Loss Factor

**Figure 9:**
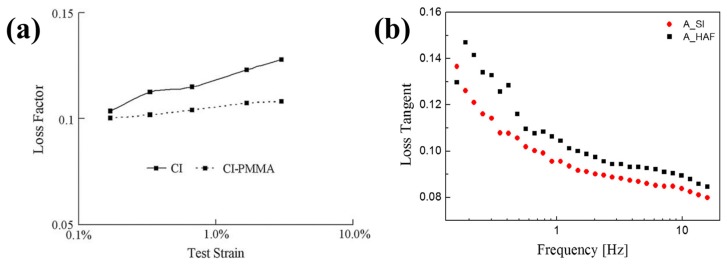
Loss factor of (**a**) CI/PMMA- (dotted) and pure CI- (solid) based MR elastomers (reprinted with permission from [75], Copyright 2009 Elsevier B. V., New York, NY, USA) and (**b**) CI/APTES- (A_SI, circle) and pure CI- (A_HAF, square) based MR elastomers (reprinted with permission from [79], Copyright 2017 Elsevier B. V., New York, NY, USA).

The loss factor is related to the bond energy between the magnetic particles and the rubber matrix. The strengthening of the bond between particles and matrix produces a small loss factor. The loss factor of magnetic particle-filled elastomer composites has three components: loss factors of elastomer matrix, filler and the interface between filler and matrix. Usually, the last component is the main source of the loss factor of composite because the bond strength between fillers and matrix is typically weak, and a kind of motion occurs between the particle and matrix. The friction caused from the motion leads to energy dissipation. Figure 9a,b shows the loss factor of both surface-coated CI and pure CI-based MR elastomers, respectively. The bond strength between fillers and the matrix of surface-coated CI-based MR elastomers is much stronger than that of the pure CI-based MR elastomers, considering the result with the lower values of the loss factor of surface-coated CI-based MR elastomers. When the magnetic field is applied to the MR elastomers, the originally dispersed magnetic particles respond to an external magnetic field even though their movement is confined within the elastomers, and the interaction force between the particles in the matrix is enhanced. With the increase of applied magnetic field, the frictional motion decreased through the strong interaction force between the magnetic particles and the elastomer matrix. As a result, the energy dissipation was reduced, and the loss factor decreased with further increasing of the magnetic field [95].

### 5.3. Tensile Strength

**Figure 10:**
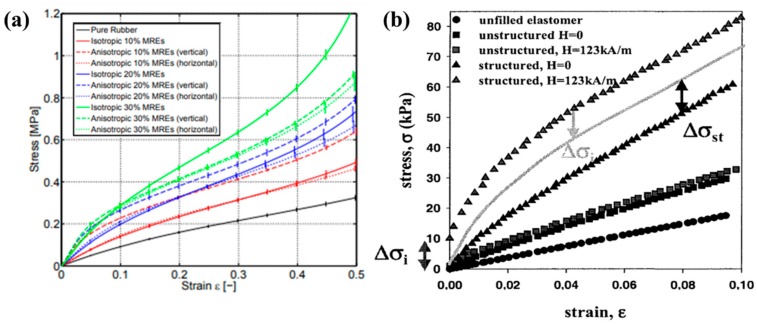
Tensile test results of pure silicone rubber, isotropic and anisotropic MR elastomers with various iron content in the absence of a magnetic field (**a**) (reprinted with permission from [124], Copyright 2015 Elsevier B. V., New York, NY, USA) and (**b**) pure silicone rubber, isotropic and anisotropic MR elastomers with 15 vol% of iron content in the presence of a magnetic field (123 kA/m) (reprinted with permission from [125], Copyright 2002 World Scientific Publishing Co. Pte Ltd., Singapore).

Based on the fact that tensile strength is one of the most important mechanical properties of rubber-based materials, it has been also widely investigated for MR elastomers. Schubert et al. [124] reported the tensile strength behavior of silicone-based MR elastomers under uniaxial tension both in the absence and in the presence of magnetic fields, as shown in Figure 10a. The tensile property increased with increasing iron particle volume concentration. The isotropic and horizontal alignment anisotropic MR elastomers showed a similar stress-strain curve shape with constantly increasing slope. In the case of vertical alignment anisotropic MR elastomers, they exhibited larger stresses than the isotropic MR elastomers except for the MR elastomer with 30% iron content. On the other hand, Chen et al. [97] fabricated the NR-based MR elastomer samples using carbon black to modify and improve their mechanical properties. The result indicated that the carbon black was able to enhance the tensile strength of the MR elastomer because the carbon black has a positive effect on the bound condition between the rubber and magnetic particles. When the magnetic field was applied on the MR elastomer, the MR elastomer became stiffer or softer than usual. Bellan et al. [125] also studied the influence of the magnetic field on the tensile strength of silicone-based MR elastomers. Figure 10b shows that the tensile strength of both isotropic and anisotropic MR elastomer was reinforced by the application of the magnetic field due to the magnetic attractive force between the particles.

## 6. Applications of MR Elastomers

The research on controlling both stiffness and damping of MR elastomers has attracted considerable interest in recent years. In addition to their active controlling capability, the fast time response of MR elastomers makes them suitable for a variety of engineering applications, such as adaptively-tuned vibration absorbers (ATVAs), isolators, sandwich beams, force sensors and actuators.

### 6.1. Vibration Absorbers

**Figure 11:**
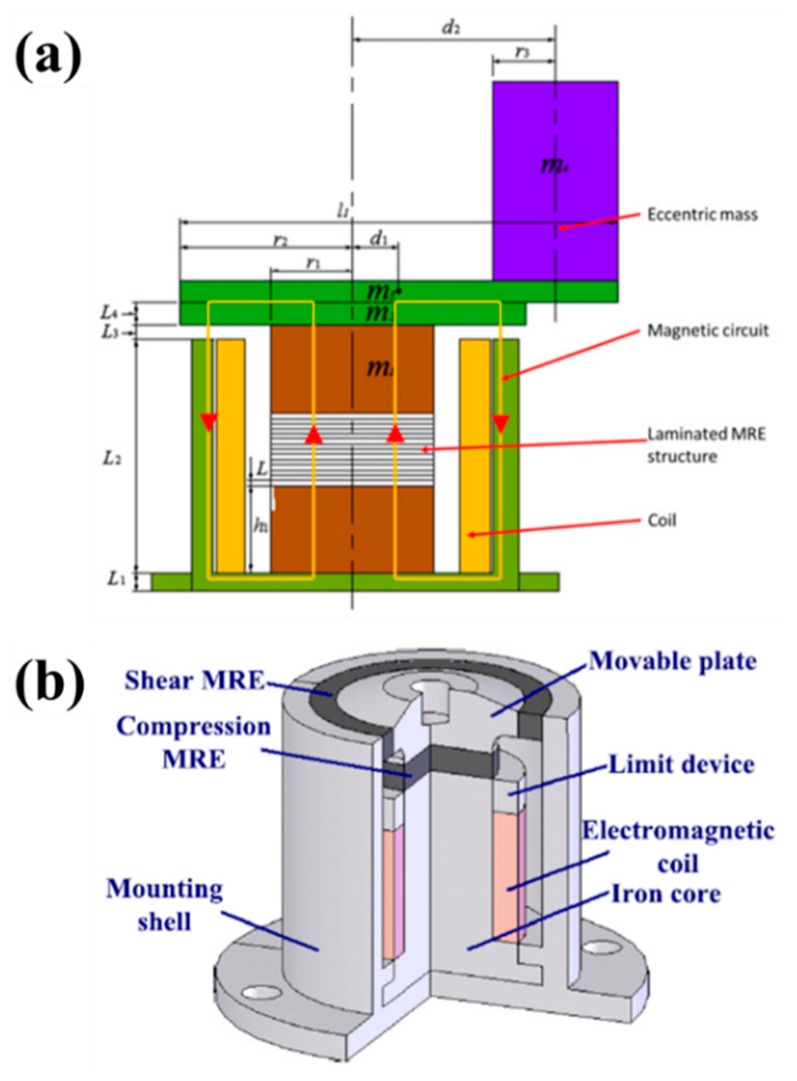
MR elastomer-based vibration absorber involving eccentric mass (**a**) (reprinted with permission from [134], Copyright 2016 IOP Publishing Ltd., Bristol, UK) and shear-compression mixed-mode MRE isolator (**b**) (reprinted with permission from [135], Copyright 2015 SAGE Publications, Thousand Oaks, CA, USA).

The unique and controllable properties of MR elastomer composites make the composites promising candidates for various applications including ATVAs. The ATVAs expand the effective band and greatly improve their performances in many applications by regulating their natural frequencies suitably to compensate for the drift in the excitation frequency. Ginder et al. [126] developed an ATVA by utilizing MR elastomers. Their experimental results indicated that the ATVA had the capability to shift frequency from 500–610 Hz. Inspired by the above work, Deng et al. [127], Sun et al. [128] and Vatandoost et al. [129] developed a variety of ATVAs in shear mode, squeeze mode and compression mode, respectively, by using MR elastomers. Williams et al. [130] did much research on ATVAs with shape memory alloys, which can vary their frequency by changing the environmental temperature. Zhou et al. [131] designed a novel smart piezoelectric actuator with controllable characteristics based on an MR elastomer. The relative change in the resonant frequency of the actuator was found to be adjusted up to 30% by applying a magnetic field. Hoang et al. [132] fabricated a conceptual ATVA with soft MR elastomers for the suppression of powertrain vibration. Their results showed that the ATVA using MR elastomer materials can effectively work in a frequency ranging from 7 Hz–70 Hz. Xin et al. [133] proposed and validated the principle of a new ATVA for powertrain mount systems of automobiles. Recently, Sun et al. [134] proposed the eccentric mass on the top of the multilayered MR elastomer structured VA for vibration reduction, as seen in Figure 11a. The vibration test results for the proposed MR elastomer illustrated that the MR elastomer absorber discerned double natural frequencies (one in the torsional direction and the other in the translational direction) that were tunable.

### 6.2. Vibration Isolators

Besides vibration absorbers, MR elastomers can also be applied to vibration isolators due to their excellent and tunable stiffness. Figure 11b shows the scheme of a shear-compression mixed mode MR elastomer isolator [135]. Two pieces of MR elastomers fabricated with different dimensions were utilized in the isolator. The test results showed that the natural frequency of the MR elastomer mixed isolator can be effectively controlled by regulating the applied magnetic field. When applying magnetic field, the natural frequency was able to increase up to 46% and 103%; the increment of stiffness was 122.15% and 329.63%; and the increment of damping was 99.07% and 180.31%. The shear MR elastomer operates in shear mode, and the other MR elastomer operates in compression mode. Behrooz et al. [136] theoretically and experimentally investigated a three-story scaled building isolated with four variable stiffness and damping isolators. Eem et al. [137] experimentally evaluated the seismic performance of a newly-proposed smart base-isolation system. Opie et al. [138] fabricated a clipped-optimal controller for an MR elastomer isolation system with 5–10-Hz excitation. Their results showed that the MR elastomer isolator system relatively reduced resonances and payload velocities by 16–30%, when compared to passive systems. Recently, Du et al. [139] reported an investigation on a semi-active/passive integrated vibration isolator by using an MR elastomer and spring. The frequency sweep test in their study illustrated that the resonant frequencies of the semi-active/passive integrated vibration isolator were able to be controlled from 30.0 Hz–51.0 Hz when the magnetic field was applied.

### 6.3. Other Applications

Bose et al. [140] designed a controllable valve utilizing soft MR elastomers. This device is able to regulate air flow through a controllable valve. Kashima et al. [141] proposed an isotropic MR elastomer soft actuator with three parts: electromagnetic coil, soft silicon rubber and MR elastomer cover. Recently, the development of an MR elastomer-based sandwich structure has been initiated, and sandwich beams have been used in various industrial areas, especially in aerospace engineering. A sandwich beam typically consists of two skin parts and a core part. Zhou et al. [142] investigated the field-dependent dynamic rigidities of sandwich beams with an MR elastomer core by finite element analysis. Nayak et al. [143] performed a dynamical analysis of the three-layer symmetrical sandwich beams with the MR elastomer embedded core and conductive layers, subjected to a periodic axial load under various boundary conditions. Ying et al. [144] examined a micro-vibration response of a clamped–free sandwich beam using an MR elastomer core and a supplemental mass under stochastic supporting micro-motional excitation. Furthermore, MR elastomers have been recently adopted for manufacturing plane electric capacitors to measure electrical capacitance [145,146].

## 7. Conclusions

In this brief review article, we provided the recent research on the fabrication, characterizations, properties and applications of various MR elastomeric composites prepared with a wide variety of magnetic particles, elastomers and additives as one example of hard and soft hybrid smart and functional materials. It was shown that the morphologies of the iso/anisotropic MR elastomers prepared with/without magnetic fields have been characterized by SEM and Fe-mapping. In addition, their magnetic properties were obtained by measuring their magnetization and hysteresis loops. The MR properties of the MR elastomer have been determined using a rotational rheometer under a range of magnetic field strengths by the strain amplitude and frequency sweep tests. The results of the MR property measurements showed that with increasing magnetic field strength, the storage moduli increased depending on the strain at a constant frequency. According to the investigation of the viscoelastic characteristics and the MR effect of MR elastomers, higher MR performance has also been observed for the anisotropic MR elastomers compared to the isotropic MR elastomers, and also, surface-coated CI particles-based MR elastomers showed higher MR performance compared to the MR elastomers with non-coated particles. Besides, the mechanical properties of MR elastomers have been characterized by the Payne effect, tensile strength and loss factor of MR elastomers. From the determination of two parameters of CI-based MR elastomers, it was found that the mechanical properties of surface-coated particle-based MR elastomers were stronger than those of pure particle-based MR elastomers because of the strengthened bonding energy between the CI particle and rubber matrix. Finally, the magnetic particle-filled elastomeric hybrid composites with controllable MR properties and fast response to external stimuli show a huge potential to be applied to a variety of engineering devices such as automotive bushings, engine mounts, adaptively-tuned vibration absorbers, isolators and actuators. It is also believed that the MR elastomeric hybrid composites can be potential candidates for biomedical devices, as well.
